# Avian Influenza Virus Surveillance in South-Central Spain Using Fecal Samples of Aquatic Birds Foraging at Landfills

**DOI:** 10.3389/fvets.2017.00178

**Published:** 2017-10-23

**Authors:** Andreia Bárbara, Olalla Torrontegi, Maria-Cruz Camacho, Marta Barral, Jose-Manuel Hernández, Ursula Höfle

**Affiliations:** ^1^SaBio Working Group, Instituto de Investigación en Recursos Cinegéticos IREC (CSIC-UCLM-JCCM), Ciudad Real, Spain; ^2^NEIKER-Tecnalia, Derio, Spain; ^3^Freelancer (Formerly affiliated with Instituto de Investigación en Recursos Cinegéticos IREC (CSIC-UCLM), Universidad de Castilla-La Mancha, Ciudad Real, Spain), Ciudad Real, Spain

**Keywords:** avian influenza virus, landfills, white storks, cattle egrets, gulls, surveillance, non-invasive sampling, H16

## Abstract

Aquatic wild birds have been intensively studied to better understand their role in avian influenza virus (AIV) maintenance and spread. To date, AIV surveillance has primarily focused on natural aquatic environments where different bird species aggregate and viral survival is enhanced. However, artificial habitats such as landfills are attracting substantial numbers of wild birds, AIV reservoir species included. The use of landfills as a predictable food source has significantly influenced population size, migratory traits, and feeding behavior of white storks (*Ciconia ciconia*) and black-headed gulls (*Chroicocephalus ridibundus*) among others. Considering the proximity of landfills to urban settlements and frequently poultry-farms, targeted monitoring of AIV in bird species that forage at landfills but are known to also frequent urban and agricultural habitats could be a useful means for monitoring of AIV, especially during periods of bird aggregation. During the wintering season 2014–2015, the prevalence of AIV in five avian species at two landfills in South-Central Spain was explored by rRT-PCR and species related temporal variation in AIV prevalence determined. We collected and tested 1,186 fresh fecal samples from white storks (*N* = 689), cattle egrets (*Bubulcus ibis, N* = 116) and mixed flocks of gulls (*N* = 381) as well as cloacal and oral swabs from five birds found dead. Seven samples contained AIV, five from gulls and one each from a stork and a cattle egret. Overall, AIV prevalence was 0.60%. No significant temporal variation was observed in AIV prevalence. Prevalence differed significantly among the sampled taxonomic groups, being highest in gulls (1.31%). H16N3 subtype was detected from a cattle egret and H11N9 subtype from a white stork, whereas gulls harbored both subtypes in addition to H11N3 subtype. H16 subtype detection in a cattle egret evidences its host range may not be restricted to gulls. Our results indicate that wild birds foraging at landfills may carry different LPAIV subtypes.

## Introduction

Avian influenza viruses (AIVs), family *Orthomyxoviridae*, genus *Influenzavirus A*, are characterized based on the properties of the hemagglutinin (HA) and neuraminidase (NA) transmembrane glycoproteins. According to pathogenicity when infecting chickens, AIV are classified into low pathogenic (LPAIV) or highly pathogenic (HPAIV) but pathogenicity varies among species, especially among wild birds (1, 2). Despite being controversial, the coincidence between LPAIV and HPAIV geographical expansion along the migratory flyways, as well as HPAIV detections in countries without previous outbreak reports in poultry, supports the theory that HPAIV can spread by means of migratory bird movements (3–5).

Avian influenza viruses circulating in natural aquatic ecosystems are generally LPAIV. Transmission occurs primarily *via* the fecal-oral route since AIV replicates mainly in the intestinal tract of birds (despite some strains may also have respiratory tract affinity) and progeny virions are shed into the environment with the feces (1). In the wild, the environmental conditions modulating AIV dynamics are very complex, and there are interacting factors that may influence detection and perpetuation of these viral particles. Because the environment provides a place for infection in different species that share the same habitats but not necessarily at the same time, its role in AIV epidemics or in sustaining viral persistence should be further investigated (6). At the same time, the exponential growth of human population as a result of the economic development of many regions, is causing natural habitat fragmentation with unprecedented perturbations of natural microbial ecology (7, 8) while on the other hand creating new artificial ecosystems. Hence, AIV dynamics in artificial ecosystems such as landfills may result in other transmission patterns of significant relevance in AIV epidemiology that need further research (9).

The Iberian Peninsula is situated along the East Atlantic and Black Sea/Mediterranean flyways of many migratory birds and its coastal and continental wetlands traditionally constitute important wintering grounds and stopover habitats for individuals on southbound migration to Africa or on return to their breeding grounds in Central and Northern Europe (10). Numbers of wintering birds in Spain have increased significantly in the past decades as many species reduce migration distance as a response to climate (11) and human-induced changes (12–14), e.g., landfills providing alternative food sources to birds.

Landfills are artificial environments that, due to constant food availability, constitute alternative stopover locations for some migratory birds and wintering quarters for others (12). Bird species foraging at landfills congregate in large flocks around recently discharged residues. While waiting for new waste trucks to arrive, these flocks rest in the surroundings of the landfill (on fields or in small ponds formed by rainfall). Although fecal-oral LPAIV transmission could be enhanced at the existing water bodies on landfills, the role of landfill habitats as a host-to-host transmission interface still remains unclear. Other pathogens of concern for public health such as bacteria carrying resistance mechanisms against antibiotics have been found to infect storks foraging at landfills (15). In Spain, the use of landfills as supplementary feeding grounds has induced a change in the migratory behavior of many species. This seems to be particularly evident in white storks (*Ciconia ciconia*) and gulls in which landfills have significantly contributed to an increase in resident individuals during the last decades (16, 17).

The Charadriiformes order is an important group among the diversity of avian species visiting landfills. This taxonomic order has traditionally been considered the second most important LPAIV reservoir after the Anseriformes. However, this assumption has recently been questioned. Reasons for this are that AIV carrier Charadriiformes are found geographically clustered in distribution and that LPAIV subtypes carried by Charadriiformes species appear to contribute more to LPAIV diversity than to maintenance of HPAIV precursor subtypes (18). Gulls (family Laridae), members of this order, are very adaptable and are found in diverse anthropic environments such as domestic poultry farms or landfills mixing with other avian species that benefit from human residues and thus could play a role as bridge species [species that due to their mobility and habitat plasticity may transmit AIV from wild reservoirs in aquatic ecosystems into domestic poultry, see Ref. (19)]. Several gull species breed inland close to lakes and marshes where they nest on the ground in large colonies (20). LPAIV frequently associated to gull species and that are rarely found in other hosts include especially H16 and H13 subtypes (21). In a study carried out on black-headed gulls in the Netherlands, LPAIV infections during the second half of the breeding season were evidenced in first year birds, with a prevalence of up to 72% per week (21). Common and abundant inland gull species in Spain include black-headed, lesser black-backed (*Larus fuscus*), and yellow-legged gulls (*Larus michahellis*). Although the breeding population of black-headed and lesser black-backed gulls in Spain is increasing, both species are largely migratory and in winter resident and wintering individuals mix with occasional yellow-legged gulls in Spanish wetlands and at landfills (22–24).

Other aquatic bird species found regularly in large numbers at landfills include white storks and cattle egrets (*Bubulcus ibis*). Storks, egrets, and herons were first grouped together in the Ciconiiformes taxonomic order. In the recent phylogenetic studies, however, herons and egrets were grouped into Pelecaniformes (25). Nevertheless, herons, egrets, and storks share similar feeding habits and breeding ecology and none are recognized as remarkably prevalent hosts of AIV (26, 27). Storks and herons are medium to large wading birds that are associated with wetland ecosystems, but in the recent past, they have adapted extremely well to the use of landfills as a constant predictable food source (12, 14). Cattle egrets are primarily resident, but white storks breeding in Western Europe are migratory and traditionally spend the winter in West Africa (28). In the recent past, however, many storks have shortened their migratory route considerably and winter on the Iberian Peninsula (14).

Since landfills act as stepping-stones in landscape connectivity between wild environments and anthropic habitats (such as farms and parks) for significant numbers of birds, samples collected at landfills may provide valuable information on AIV epidemiology.

For this reason, we utilized two landfills in South-Central Spain to study AIV prevalence dynamics through autumn and winter, in selected aquatic species that use these artificial environments for foraging. We focused on temporal patterns such as month and bird phenology and host species variation to monitor potential AIV prevalence and subtype fluctuation.

## Materials and Methods

### Study Area and Period

The study was carried out at two landfills located in South-Central Spain in the Autonomous region of Castilla-La Mancha (Alcázar de San Juan 39°26′N 3°13′E and Almagro 38°51′N 3°39′E). This area corresponds to the climatically and geographically defined bioregion three as described previously (29). Both landfills share geographical proximity with natural aquatic ecosystems (Tablas de Daimiel National Park, 29.9 km and Vegas del Jabalón dam 11.1 km flying distance from Almagro landfill, and the La Veguilla, Yegua, and Camino de Villafranca lagoons comprising wetland at 5 km flying distance from the Alcázar de San Juan landfill) and human settlements (4.8 km and 5.7 km flying distance from Alcázar de San Juan and Almagro town centers, respectively). In the case of the Almagro landfill, there are also at least a couple of poultry farms in a radius of less than 10 km.

**Figure 1:**
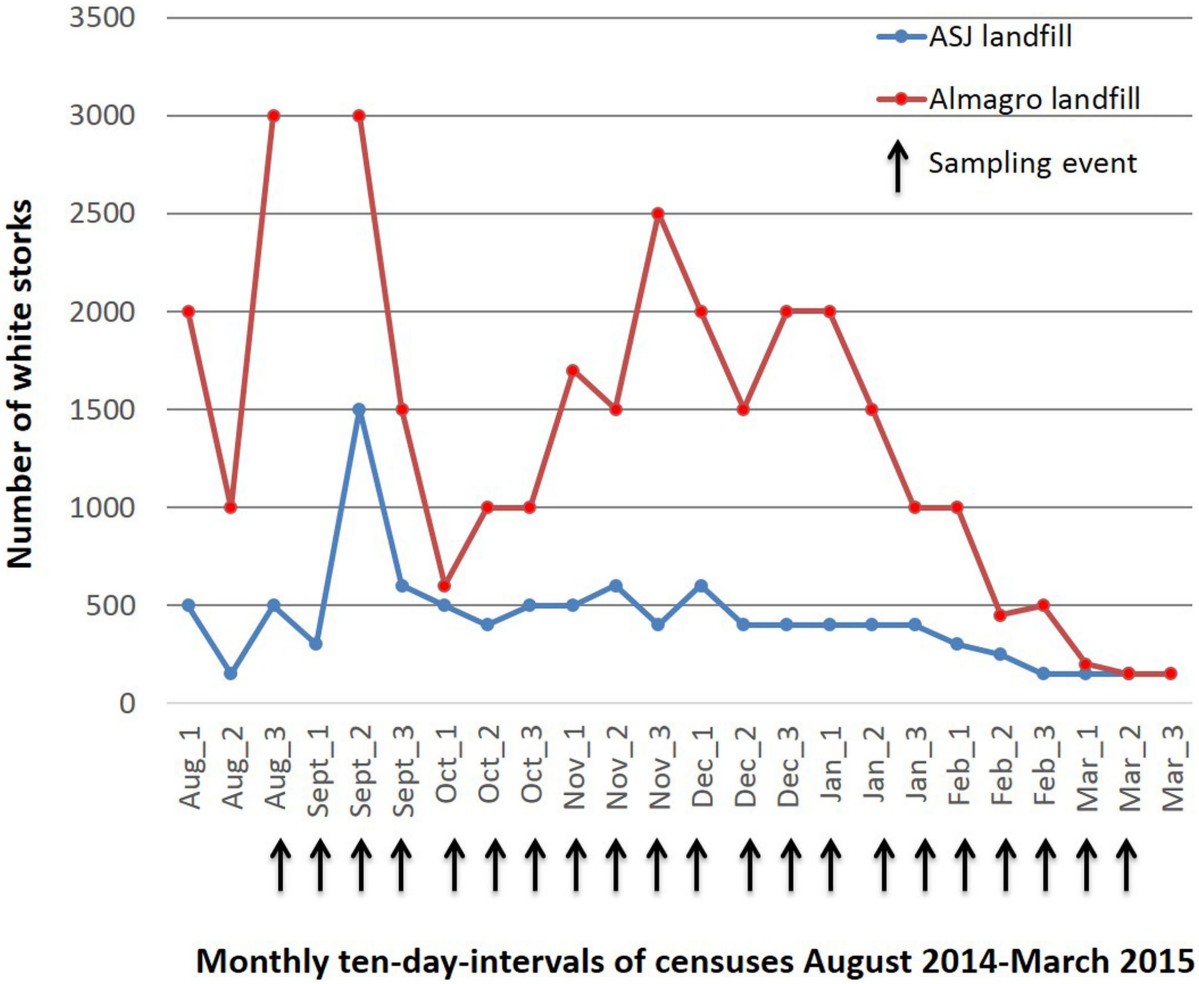
Approximate numbers of White storks foraging at the Alcázar de San Juan and Almagro landfills between August 2014 and March 2015.

Both landfills have been closely followed for use by white storks since august 2011 and approximate counts of total number of white storks and ring code reads have been carried out every ten days between August 2012 and March 2016 (unpublished data). During the migratory and wintering period (August to March), total numbers of white storks increase four to 10-fold at the Alcazar de San Juan landfill and 10- to 20-fold at the Almagro landfill (Figure 1). Other bird species foraging at the landfills in large numbers included black-headed, lesser black-backed and yellow-legged gulls, cattle egrets, European starlings (*Sturnus vulgaris*), and temporarily black kites (*Milvus migrans*). The Alcazar de San Juan landfill holds a small colony of white storks (seven nests) and a sleeping roost of cattle egrets of approximately 50 individuals on its premises. Fewer numbers of gray herons (*Ardea cinerea*), wagtails (*Motacilla alba*), and many other passerines could be observed at the landfill.

Due to the serious decline and local extinction of the white stork in Southern, Central, and Northern Europe during the early twentieth century, numerous reintroduction programs and ringing schemes have been in place for decades for this species across Europe that largely employ rings for visual recapture (28). For the sampled landfills, white stork ring-readings have been carried out since 1996 and as described, more intensively between 2011 and 2016. Visual recapture data reveal the use of the sampled landfills for stopover on southward and northward migration as well as wintering by white storks originating from central Europe as well as by local residents (data not shown). It is very likely that a similar situation applies to the different gull species that compose the mixed gull flocks at the landfills, especially for the black-headed and lesser black-backed gull. This assumption is further supported by literature based on ring recoveries (22), GPS satellite data (23), and census data of breeding and wintering populations of the three gull species for Spain (24). In contrast, cattle egrets in Europe are primarily non-migratory; thus, we assume that most of the individuals observed are resident in the area.

The selection of species for sampling was based upon accessibility for fresh fecal droppings for collection, as it was not possible to sample individuals that were foraging actively. The species selected for sampling in this study were those that were observed in groups of significant numbers close to the areas where rubbish was deposited, around small ponds or puddles forming on or close to the landfill premises or on fields surrounding the landfill and that could be observed moving between the residues and the resting areas. Selected species included white storks, cattle egrets and the three gull species; black-headed gulls, lesser black-backed gulls, and yellow-legged gulls.

Based upon the mean duration of LPAIV infections in anatids and gulls reported in the literature (21, 30), sampling was performed every 15 days, between September 2014 and March 2015 in order to study AIV prevalence dynamics during stopover of migrating birds on southward migration (September), wintering (October to December), and stopover on northward migration (January to March) of white storks, lesser black-backed gulls, and black-headed gulls in Spanish territories. This resulted in a total of 26 sampling visits (14 at the Alcazar de San Juan landfill and 12 at the Almagro landfill as administrative constraints delayed access permission).

### Sampling

In order to minimize the risk of sampling the same bird more than once, only fresh feces were collected at each landfill-site out of the landfill body from flocks of single species after flushing them by approaching. Gull species, however, usually were present in mixed flocks. Therefore, the three gull species were considered as a single flock unit for sampling (family Laridae). Prior to approaching the flocks, an approximate count of the flock size was attempted, which however was limited in accuracy especially for gulls as frequently only part of the flock was visible due to ditches or similar landscape features. When possible, sample size was adapted to flock size based upon the mean AIV prevalence found previously in the area (26); when not, approximately 30 samples were taken from each flock unit every sampling time. As cattle egret flocks were occasionally considerably smaller, fewer samples were available for collection. On some sampling visits, cattle egrets were not present altogether outside the residue foraging area and could not be sampled. Samples were individually taken from the environment with a sterile cotton swab and placed inside a small zip-lock bag. They were maintained refrigerated (4–10°C) during transport to the laboratory facilities. In the laboratory, a swab sample of each dropping was collected individually in a container with viral transport medium [Hank’s balanced solution containing 10% glycerol, 200 U/ml penicillin, 200 mg/ml streptomycin, 100 U/ml polymixin B sulfate, 250 mg/ml gentamicin, and 50 U/ml nystatin (31)]. Consecutively, five unit samples were pooled (according to the flock species and landfill) into a single container.

In addition to fecal samples, carcasses of recently dead black-headed gulls (*N* = 2) or sick lesser black-backed gulls (*N* = 1), black-headed gulls (*N* = 1), and white stork (*N* = 1) found at the landfills during sampling visits were also collected and sampled. These samples consisted of one cloacal and oral swab per specimen. Both swabs from each dead or sick animal were pooled and individually processed for each case.

Viral RNA was extracted using a commercial kit (High PureRNA isolation kit, Roche Diagnostics, Germany), according to the manufacturer’s instructions. 200 µl transport medium were used for eluting 50 µl RNA. RNA was quantified using NanoDrop 1000 Spectrophotometer V3.7 (Thermo Fisher Scientific, Wilmington, DE, USA) and screened following an rRT-PCR (rRT-PCR) protocol targeting the Influenza A virus matrix gene (31). Amplification and detection was performed using an iQ5 real time detection system (BioRad, Applied Biosystems, NJ, USA) for all the rRT-PCR assays. When a pool tested positive RNA extraction and screening by rRT-PCR was repeated for the individual samples composing the pool. Further testing (H5/H7 subtype PCRs and viral isolation) was only carried out for the individual positive sample.

Avian influenza virus positives (AIV+) were also analyzed for H5 and H7 subtypes by rRT-PCR (31, 32). All AIV-positive samples were submitted for viral isolation and sequence analysis. 100–200 µl of the original fecal material was inoculated into the allantoic cavity of 9–11 day-old embryonated SPF chicken eggs following OIE recommendations (33). The allantoic fluid was harvested after death of the embryo or at the 7th day after inoculation. RNA was extracted using a commercial kit (QIAmp Viral RNA Mini kit, Qiagen, Hilden, Germany) and M gene specific rRT-PCR for AIV detection (32). If no AIV was detected, the allantoic fluid was passaged twice in a new set of embryonated chicken eggs. AIV+ HA and neuraminidase subtyping was also performed (34, 35).

We compared AIV prevalence (%, with 95% Wilson score confidence interval) among species, sampling months and migratory periods using Fisher’s exact test. Statistical analysis was performed with SAS^®^ 9.3 statistics software.

## Results

Between September 2014 and March 2015, a total of 1,186 fresh fecal samples were collected, from white storks, gulls, and cattle egrets (Table 1). Global AIV prevalence was 0.60% [(0.30–1.21); *N* = 7/1,186]. None of the samples taken from dead or diseased animals (*N* = 5) was AIV-positive.

**Table 1:** AIV prevalence in white storks, cattle egrets, and gulls sampled in two landfills in South-Central Spain.

	*Ciconia ciconia*		*Bubulcus ibis*		Laridae		Total	
Variables	AIV+/*N*	% Prev (CI)	AIV+/*N*	% Prev (CI)	AIV+/*N*	% Prev (CI)	AIV+/*N*	% Prev (CI)
Taxa*								
	1/689	0.15 (0.03–0.82)	1/116	0.86 (0.15–4.72)	5/381	1.31 (0.56–3.03)	7/1186	0.60 (0.30–1.21)
AIV subtype	H11N9		H16N3		H16N3 × 2, H11N9, H11N3			
**Month**								
September-2014	0/26	0.00 (0.00–12.87)	0/10	0.00 (0.00–27.75)	0/0	0.00 (0.00–0.00)	0/36	0.00 (0.00–9.64)
October-2014	0/98	0.00 (0.00–3.80)	1/35	2.86 (0.51–14.53)	3/75	4.00 (1.37–11.11)	4/208	1.92 (0.75–4.84)
November-2014	0/91	0.00 (0.00–4.05)	0/30	0.00 (0.00–11.35)	0/46	0.00 (0.00–7.71)	0/167	0.00 (0.00–2.25)
December-2014	0/135	0.00 (0.00–2.77)	0/34	0.00 (0.00–10.15)	1/69	1.45 (0.26–7.76)	1/238	0.42 (0.07–2.34)
January-2015	1/127	0.80 (0.14–4.33)	0/0	0.00 (0.00–0.00)	0/71	0.00 (0.00–5.13)	1/198	0.50 (0.09–2.80)
February-2015	0/126	0.00 (0.00–2.96)	0/7	0.00 (0.00–35.43)	1/60	1.67 (0.30–8.86)	1/193	0.52 (0.09–2.87)
March-2015	0/86	0.00 (0.00–4.30)	0/0	0.00 (0.00–0.00)	0/60	0.00 (0.00–6.02)	0/146	0.00 (0.00–2.60)
**Migratory period**								
Southward migration	0/26	0.00 (0.00–12.87)	0/10	0.00 (0.00–27.80)	0/0	0.00 (0.00–0.00)	0/36	0.00 (0.00–9.64)
Wintering	0/324	0.00 (0.00–1.17)	1/99	1.01 (0.18–5.50)	4/190	2.11 (0.82–5.30)	5/613	0.82 (0.35–1.89)
Northward migration	1/339	0.30 (0.05–1.65)	0/7	0.00 (0.00–35.43)	1/191	0.52 (0.09–2.90)	2/537	0.37 (0.10–1.34)

*N, number of collected samples; AIV, number of AIV-positives in rRT-PCR and AIV subtypes; CI, 95% confidence interval*.

*Significant variables with p < 0.05.

There were significant differences in AIV prevalence among the sampled species (*p* = 0.04); gulls [1.31% (0.56–3.03); *N* = 5/381], cattle egrets [0.86% (0.15–4.72); *N* = 1/116], and white storks [0.15% (0.03–0.82); *N* = 1/689] (Table 1). Pair-wise comparisons revealed significant differences in AIV prevalence between gulls and white storks (*p* = 0.02).

As for the migratory periods, AIV prevalence was 0.82% [(0.34–1.90); *N* = 5/613] and 0.37% [(0.10–1.34); *N* = 2/537] during the wintering season and northward migration, respectively, but no virus was detected during the southward migration. Except for gulls, all species were sampled during all migratory periods (Table 1). Although AIV prevalence peaked in October [1.92% (0.80–4.83); *N* = 4/208], differences in prevalence were not significant neither monthly nor seasonally (Table 1).

All AIV-positives were LPAIV. Viral recovery rate was 66.67% (4/6). For one of the samples, material was insufficient for culture. In two of the three positive samples, in which, virus isolation was unsuccessful subtype could be directly determined by rRT-PCR. Thus, 6 out of the 7 positive cases belonged to either H11 or H16 subtypes. Gulls harbored H11N3, H11N9, and H16N3 (×2) subtypes. The positive sample from a cattle egret belonged to the H16N3 subtype and the white stork carried H11N9.

## Discussion

The present study reports, for the first time, AIV in aquatic birds that forage at Spanish landfills. Overall LPAIV prevalence was low (0.60%), which could be related to the species sampled, the time of the year chosen for the study or the type of sample.

Generally, AIV prevalence is highly variable in natural ecosystems depending on location, time of the year and host species targeted (36–38). In addition, high inter-annual variability causes significant fluctuation in AIV prevalence. In the previous studies conducted in natural environments in Spain, LPAIV prevalence varied between 2.58% (2005–2007) and 5.00% (2006–2009) in Castilla-La Mancha (South-Central Spain) and Catalonia (North-Eastern Spain), respectively (26, 38). In addition, a third study performed between 2007 and 2009 at wetlands from Castilla-La Mancha, Catalonia, and the Basque Country, found a 1.70% overall AIV prevalence (36). Albeit, in the latter authors found important fluctuations in prevalence between years, varying from 0.82 to 7.67%. In this regard, the low prevalence detected in this study may mean that the study has covered a period of low prevalence in the area.

The low AIV prevalence detected in our study may also be due the absence of samples from Anatidae, the wild bird family with highest LPAIV prevalence (39). As aquatic herbivores, this taxonomic group does not seem to be attracted by landfills to complement their feed intake and thus could not be included in this study (1). Nevertheless, although LPAIV is generally detected in members of the Anatidae family, occasionally also in this family prevalence may be low, such as for example, during 2012–2014 (0.29%) in a recent long-term study carried out in a Spanish wetland.^1^ Taking a closer look at the species included in the study, in a previous study using the same method (fresh fecal samples) in natural wetlands situated in the same area (South-Central Spain), both white storks and cattle egrets tested positive for AIV. In both cases, prevalence was higher (0.78 and 1.36% for white storks and cattle egrets, respectively), while no AIV was detected in gulls which in this case was thought to be due to low sample size (26).

Gulls were found to excrete AIV more frequently and harbored more subtypes than the rest of the species tested. This is in agreement with previous studies pointing out at Charadriiformes as AIV reservoirs in natural environments (1) (Table 1). Interspecies variation in AIV prevalence may be due to intrinsic differences in host susceptibility and ecology (2, 40). In this regard, gulls were more frequently found at the aquatic bodies of the monitored landfills where viral persistence may be enhanced. Because of the geographical proximity of both landfills to local wetlands, the possibility of these natural aquatic ecosystems to be the source for AIV infection should also be considered.

Previous studies have suggested that the H16 AIV subtype is endemic to gulls (41–44). However, the H16N3 subtype detection from a cattle egret may indicate a larger host range. Despite the low prevalence of AIV in cattle egrets a potential role for these in AIV epidemiology should not be disregarded. Targeting cattle egrets and other species in significant numbers for AIV detection might shed more light on their role in AIV maintenance and spread in the context of AIV as multi-host system as has been suggested recently (18). White storks in contrast to cattle egrets have been tested extensively locally with mostly negative results (45), suggesting infections in white storks are more likely related to spillover events from other species such as gulls. H16 AIV subtypes are not considered a risk for domestic poultry as they are, together with H13 mostly found in gulls. However, a recent study has reported isolation of H11 AIV subtypes from domestic poultry and evidenced efficient replication and transmission in chickens (46).

The selection of the species included in this study was based upon abundance, previous AIV detection in the species and accessibility for sampling without the necessity of capture. All of the species are also closely related to anthropic habitats conferring them thus a potential role as bridge species in AIV transmission to domestic animals (4). Consistent census data for the study period are only available for white storks at both study sites, showing a significant four to twentyfold increase in white stork numbers during the migratory and wintering season (Figure 1), with a considerable proportion of storks originating from Central or Northern Europe (data not shown) as identified by visual recapture. While numbers of resident cattle egrets remained largely constant, observational data from the two landfills showed a significant increase in numbers during the migratory and wintering periods also for black-headed and lesser black-backed gulls, accordingly to the phenology of migrating and wintering birds at natural wetlands that constitute significant stopover or wintering areas (24, 26). White storks are traditionally long distance migrants with wintering grounds in Central or Southern Africa, but a number of factors have led to behavioral changes with a large proportion of storks now wintering in the Iberian Peninsula, but their inland resident populations have also recently increased (14). Black-headed and lesser black-backed gulls, and to much lesser degree yellow-legged gulls traditionally winter in the Iberian Peninsula, but have also recently increased inland resident populations, also mostly due to the use of landfills (22–24). Taken together, landfills constitute a new human-made habitat that allows close contact of a number of species many of which have not been studied sufficiently to understand there potential role in AIV epidemiology (18). Also they create new bird communities (a specific selection of species in interaction based on the type of habitat provided by the landfill environment) that could create unique epidemiological scenarios for AIV. Hence, leading to new strain exchanges, recombination and a new role in AIV maintenance and spread to poultry or humans for the implied species. In this context, our study should also have included starlings that are present in large flocks at the landfills; however, sampling strategy for this species probably would need to involve capture.

Similar to many AIV surveillance studies in natural wetlands, our study was designed to detect LPAIV circulation during the migratory and wintering period (39, 44). Sampling during a full annual cycle might have allowed for differentiation between the importance of resident and migratory individuals in the maintenance of AIV in the landfill habitat. Both gulls and white storks initiate autumn migration on their breeding grounds in late summer (22, 23, 47), which is when the majority of AIV positive individuals are detected (39). Negative results for the autumn migration stopover period in our study may have resulted from low sample size and a late start in sampling (September) and the failure to collect samples from gull flocks in our first sampling visits. The prevalence peak observed in October although not significant matches the results observed in an earlier study on AIV prevalence in fecal samples in birds from natural wetlands in South-Central Spain (26) and may correspond to the arrival of wintering birds that remain at the landfills and circulation between these and resident birds (cattle egrets).

Fecal sampling does not allow to identify the specific individual shedding AIV. Thus, whether detections are associated to resident birds or migrants could not be determined. Sampling resident and migratory individuals from partially migrant species (when a fraction of the metapopulation is migratory and the other sedentary) would have provided key data on the contribution of each of the subpopulations. In natural ecosystems, the mallard duck (*Anas platyrhynchos*) is one of those partially migrant species in which AIV infections are known to be bidirectional between migratory and resident members (48). It has been proposed that post-breeding migrants arriving into new grounds may allow the introduction and circulation of new viral strains, while becoming infected by AIV subtypes maintained by resident birds (48–50). As foraging at landfills is focused on discharge of newly arrived rubbish brought in by trucks, many birds spend long intermediate resting periods as flocks on, or close to the premises of the landfills, creating optimal conditions for fresh fecal sampling. Even if non-invasive techniques do not detect oral viral shedding, fresh fecal sample collection has been described as an appropriate method for large-scale LPAIV surveillance programs in wild birds as it is cost-effective and causes little impact in the wild bird community (26). However, it may lead to an underestimation of AIV prevalence as the combined analysis of oral and cloacal swab samples has been shown by several studies to yield more AIV detections than cloacal or fecal sampling alone (51).

In summary, the present study identified circulation of AIV in aquatic bird species foraging at the studied landfills evidencing that these places may also act as appropriate environments for AIV transmission. AIV was detected in all studied species, demonstrating they were all susceptible to an AIV infection. Higher AIV detection rates in gulls are in agreement with findings of other authors and reflect that gulls may be important in the maintenance of AIV epidemiology as stated previously (4, 21). As described in other studies, the arrival of migrating wild birds during the wintering season to Spain could be associated with a peak in AIV detection within wild bird populations foraging at landfills. Thus landfills could be relevant in AIV epidemiology providing new AIV transmission pathways between different bird species and as a link at the wildlife-human interface. The detection of H11 subtype AIV that may be able to spread into domestic poultry and also potentially infect humans in addition to the high concentration of birds landfills harbor should render these convenient places for routine AIV surveillance in potential bridge species and for anticipation to future outbreaks, considering their proximity to urban settlements and to poultry farms.

## Author Contributions

AB, MC-C: carried out field sampling and laboratory analysis, compiled the data, and drafted the first version of the manuscript. OT: carried out laboratory and data analysis and interpretation, wrote the manuscript. J-MH: acquired, compiled, and analyzed field data, critically revised the manuscript. MB and UH: conceived and designed the study, revised the data analysis, and wrote and revised the manuscript.

## Conflict of Interest Statement

The authors declare that the research was conducted in the absence of any commercial or financial relationships that could be construed as a potential conflict of interest. The reviewer AC and handling editor declared their shared affiliation.

## Footnotes

^1^Torrontegi O, Álvarez V, Acevedo P, Gerrikagoitia X, Höfle U, Barral M. Long-term avian influenza virus epidemiology in a small Spanish wetland ecosystem is driven by the breeding Anseriformes community. J Appl Ecol Under Rev (2017).
